# Temperature and moisture are minor drivers of regional-scale soil organic carbon dynamics

**DOI:** 10.1038/s41598-019-42629-5

**Published:** 2019-04-23

**Authors:** B. González-Domínguez, P. A. Niklaus, M. S. Studer, F. Hagedorn, L. Wacker, N. Haghipour, S. Zimmermann, L. Walthert, C. McIntyre, S. Abiven

**Affiliations:** 1 Department of Geography, Soil Science and Biogeochemistry Unit, University of Zurich (UZH), Winterthurerstrasse 190, 8057 Zurich, Switzerland; 2 Department of Evolutionary Biology and Environmental Studies, University of Zurich (UZH), Winterthurerstrasse 190, 8057 Zurich, Switzerland; 3 Forest Soils and Biogeochemistry, Swiss Federal Institute for Forest, Snow and Landscape Research (WSL), Zürcherstrasse 111, 8903 Birmensdorf, Switzerland; 4 Department of Physics, Laboratory of Ion Beam Physics, Swiss Federal Institute of Technology (ETH), Otto-Stern-Weg 5, 9083 Zurich, Switzerland; 5 Institute of Geology, Department of Earth Sciences, Swiss Federal Institute of Technology (ETH), Sonneggasse 5, 8092 Zurich, Switzerland; 6 AMS Laboratory, Scottish Universities Environmental Research Centre (SUERC), Rankine Avenue, G75 0QF East Kilbride, UK

**Keywords:** Carbon cycle, Carbon cycle

## Abstract

Storing large amounts of organic carbon, soils are a key but uncertain component of the global carbon cycle, and accordingly, of Earth System Models (ESMs). Soil organic carbon (SOC) dynamics are regulated by a complex interplay of drivers. Climate, generally represented by temperature and moisture, is regarded as one of the fundamental controls. Here, we use 54 forest sites in Switzerland, systematically selected to span near-independent gradients in temperature and moisture, to disentangle the effects of climate, soil properties, and landform on SOC dynamics. We estimated two SOC turnover times, based on bulk soil ^14^C measurements (τ_14C_) and on a 6-month laboratory soil incubation (τ_i_). In addition, upon incubation, we measured the ^14^C signature of the CO_2_ evolved and quantified the cumulated production of dissolved organic carbon (DOC). Our results demonstrate that τ_i_ and τ_14C_ capture the dynamics of contrasting fractions of the SOC continuum. The ^14^C-based τ_14C_ primarily reflects the dynamics of an older, stabilised pool, whereas the incubation-based τ_i_ mainly captures fresh readily available SOC. Mean site temperature did not raise as a critical driver of SOC dynamics, and site moisture was only significant for τ_i_. However, soil pH emerged as a key control of both turnover times. The production of DOC was independent of τ_i_ and not driven by climate, but primarily by the content of clay and, secondarily by the slope of the site. At the regional scale, soil physicochemical properties and landform appear to override the effect of climate on SOC dynamics.

## Introduction

Earth System Models (ESMs) have become a primary tool to project future climate^1^. Given that soil organic carbon (SOC) is one of the largest terrestrial C pool (~3000 Pg C)^2^; and relevant to terrestrial carbon-climate feedbacks, the adequate representation of soils is critical for these models. SOC dynamics is commonly implemented in ESMs as a function of climate^3,4^, typically represented by the interplay of temperature and moisture. Soil incubations and short-term (<10 years) field studies have generally indicated that SOC reservoirs are vulnerable to warming^5,6^. Moisture has also been recognized as important for predicting the evolution of SOC stocks and fluxes, but its influence has been less thoroughly researched compared to temperature^7,8^. The evaluation of well-established ESMs has shown that they yield widely different projections of SOC stocks and dynamics and that their results poorly fit observations^9,10^. A major cause of uncertainties relates to the global carbon cycle^9^ and it has been suggested that they could be partly reduced if the simulation of SOC turnover was improved^1,10^. For this, the dependences of SOC dynamics on key drivers (e.g. climate, clay content) should be better understood and implemented in carbon models^10,11^.

**Figure 1:**
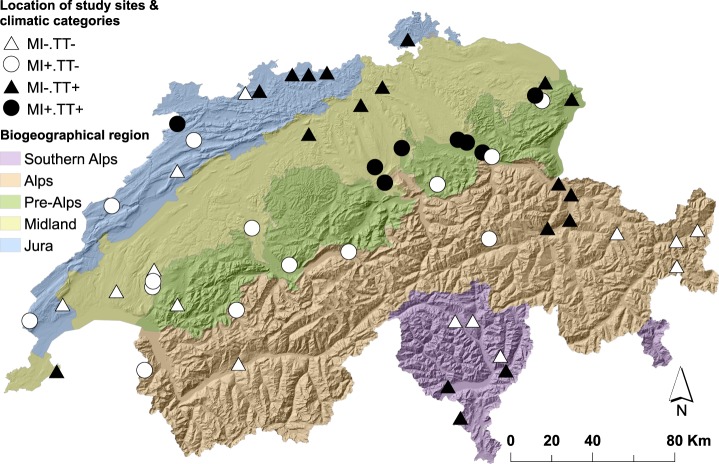
Geographic location of the 54 forest study sites selected to maximize the orthogonality of the putative drivers of SOC dynamics. The shape and colour of the points refer to the climatic category and align with Fig. 2. Description of climatic categories: MI- dry site, MI+moist site, TT− cold site and TT+ warm site. Swiss biogeographical regions are indirectly connected to biogeophysical characteristics. Map prepared in ArcGIS 10.5 (www.esri.com/arcgis).

A common limitation of observational studies on this topic is the inherent covariation of drivers in time and/or space (e.g. site temperature and moisture, precipitation and soil pH), as this covariation across natural gradients makes it very challenging to disentangle the effect of drivers^12–14^. The complex orography of Switzerland, with the Alps separating the northern and southern regions, generates a large diversity of climates ranging from artic to mediterranean. To unravel the regional-scale effect of temperature and moisture on SOC dynamics, we developed a statistics-based strategy to select 54 study sites spread over Switzerland with maximized orthogonality of temperature and moisture (Fig. 1). We collected field data, ran a laboratory soil incubation and measured ^14^C contents to test concurrently various putative drivers of SOC dynamics. Our main hypothesis was that climate plays a primary role, followed by soil properties and landform.

We selected the 54 study sites from the forest soils database of the Swiss Federal Institute for Snow, Forest and Landscape Research (WSL) containing information on 1,050 profiles^15^ (Figs 1 and S4). To represent climate, we included site temperature (1981–2010 average air mean monthly temperature) and moisture (1981–2010 dryness index, details in Methods). For soil properties and landform, we gained pH, clay content (%), slope (%) and orientation (N-E-S-W) data and reduced them to two orthogonal variables (PCo1, PCo2) by principal coordinate analysis. As the Swiss biogeographical regions^16^ (Fig. 1) differ in biogeophysical characteristics that may also affect SOC stocks and turnover rates, we fitted them as an independent block factor during data analysis. The 54 study sites were selected so that the effects of biogeographic region, temperature, moisture, and the aggregated soil properties and landform-related variables (PCo1, PCo2) were near-orthogonal, i.e. their effects were independent in the multiple linear regression models summarized by analysis of variance^17,18^ (details in Methods).

From each of the 54 sites, we collected three mineral soil samples from three non-overlapping areas of a plot (~1,600 m^2^). Each of these samples was a composite obtained by mixing eight 0–20 cm depth soil cores. We determined the bulk soil radiocarbon signature by accelerator mass spectrometry^19^ on a mixture of material from these three bulk soil composites. Further, we incubated fresh soil samples (sieved ≤2 mm) in microlysimeters, which permitted the flushing of the soils during the incubation to yield water extracts^20^. For 181 days, we repeatedly measured SOC mineralisation as the evolution of CO_2_ and the production of dissolved organic carbon (DOC) by determining organic carbon in soil water extracts. We measured ^14^CO_2_ evolved upon incubation by accelerator mass spectrometry. We ran the incubation in the dark at constant temperature and moisture (25 °C and water potential of −20 kPa).

SOC dynamics reflects the balance between the long-term above and belowground organic carbon (OC) inputs, as well as SOC loss processes. Assuming system homogeneity, we calculated two separate estimates of turnover time and compared them. First, bulk soil ^14^C measurements were used to calculate turnover time τ_14C_, based on near steady-state conditions (i.e. inputs ≈ outputs) and not accounting for lag times^21^. Second, SOC mineralisation data from the laboratory soil incubation was used to estimate turnover time τ_i_ as the ratio of total SOC stock and output flux^22^ (details in Methods). As the dissolution of organic substrates is a requirement for their transfer across microbial membranes^23^, sorption to minerals^24,25^, and large-scale transport across ecosystems and export from soils^26,27^, DOC constitutes a small but decisive component of the SOC cycle. Thus, we also quantified the DOC flux as the cumulative production throughout the 181-day incubation.

## Results and Discussion

### Turnover of contrasting ‘fractions’ of the SOC continuum

**Figure 2:**
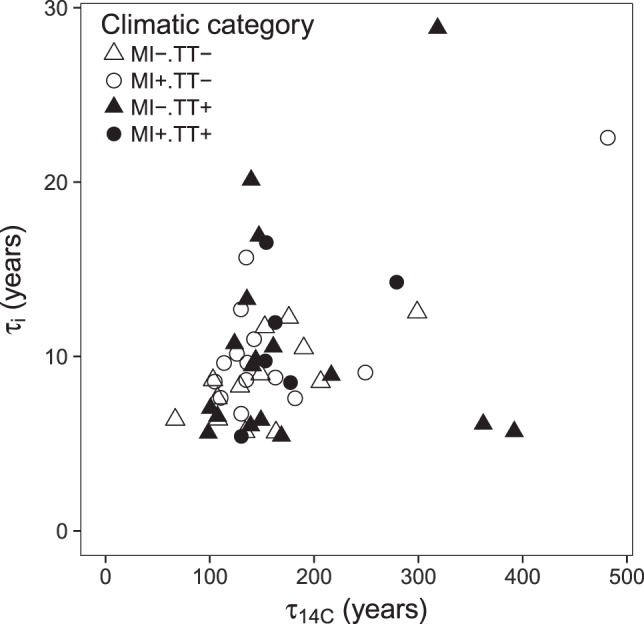
Correlation between turnover time estimates (years) from radiocarbon (τ_14C_) and a laboratory soil incubation (τ_i_) data. One site was excluded from the analysis of τ_14C_ and three from the analysis of τ_i_, due to unrealistic value and saturation of NaOH traps respectively (details in Methods). Description of climatic categories: MI− dry site, MI+moist site, TT− cold site and TT+ warm site.

**Figure 3:**
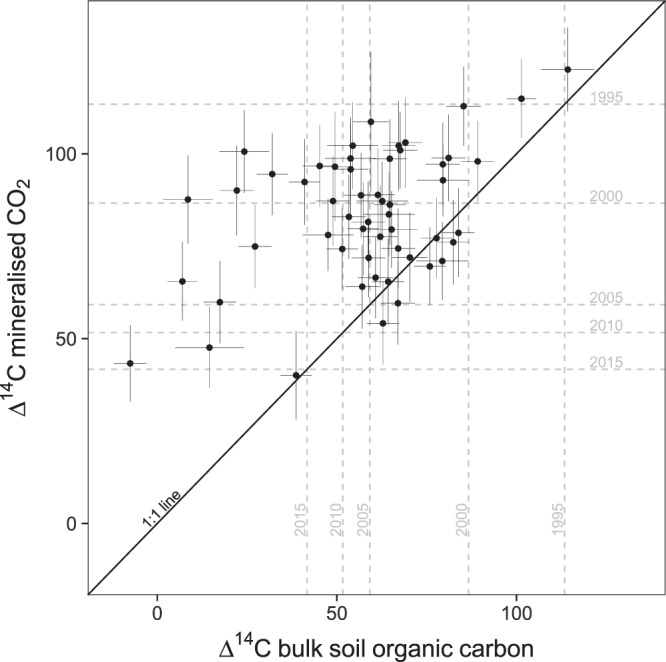
Radiocarbon signatures of bulk soil organic carbon and CO_2_ evolved during the last 31 days of a 181-day laboratory soil incubation (0–20 cm depth mineral soil) (Fig. 1). Points are Δ^14^C measured values corrected for 2014, year of field sampling. Uncertainty associated with Δ^14^C bulk soil organic carbon are analytical errors and to Δ^14^C mineralised CO_2_ are based on a mixing model assuming constant contamination^68^. Grey discontinuous lines represent atmospheric ^14^CO_2_ (North Hemisphere zone 2^75^) and serve as a reference to approximate when the CO_2_ evolving from the samples was mostly fixed by vegetation. Values > 0 indicate the presence of Bomb ^14^C; values < 0 indicate that samples have been isolated from atmospheric exchange long enough for considerable radioactive decay to occur and values = 0 indicate that the signature of the samples is equal to the one of the 1890 wood standard which represents pre-industrial atmospheric ^14^CO_2_.

Our data show that the ^14^C-based τ_14C_ is at least one order of magnitude larger than the incubation-based estimate τ_i_ (Fig. 2), reinforcing the marked discrepancy found between both calculation methods in a review by Feng *et al*.^28^. The ^14^C-based τ_14C_ ranges from 67 to 511 years and represents the average turnover time of the mixture of all OC molecules that constitute bulk SOC^29^. In general, τ_14C_ reflects SOC cycling on time scales ranging from years to millennia, which in mineral soils typically constitutes most of the stock^30^ and where SOC stabilisation processes prevail. As the terrestrial carbon cycle does not only respond to a mean climate, but also to a time series of weather conditions and extreme events^31–33^, the long-term nature of τ_14C_ makes this metric suited to capture the cumulative effect of such events. On the other side, the incubation-based τ_i_, based on the OC output flux over the course of the incubation, ranges from 5 to 29 years, in agreement with the radiocarbon signature of the CO_2_ evolved, which carries the atmospheric ^14^C signature of the last ~5–20 years (Fig. 3). Therefore, τ_i_ primarily but not exclusively, reflects plant-derived readily available OC^34^ not yet stabilised by mineral interactions or occlusion within aggregates^35^. In addition, our estimates of τ_14C_ and τ_i_ comply with the concepts of *mean age* and *mean transit time*, respectively. Sierra *et al*. (2016) presented these metrics to contribute to resolving the misconception in literature between the terms turnover time, age and residence time. Further, the discrepancy between the radiocarbon signature of the bulk soil and of the CO_2_ evolved (Fig. 3) evidences the heterogeneity of the soil systems. In fact, in a truly homogeneous system τ_14C_ would equal τ_i_ (i.e. mean age = mean transit time). Quantitatively, during the 181-day incubation, soils lost 1–9% OC as CO_2_ and DOC, indicating that under perturbation a labile fraction of SOC will be potentially lost rapidly.

Todd-Brown *et al*.^10^ simulated global SOC turnover for the period 1995–2005 with ESMs part of the Coupled Model Intercomparison Project phase 5 (CMIP5). As many of these models do not explicitly report the depth of OC in the soil profile, the authors assumed that simulations referred to the top 1 m. Their calculated turnover times ranged between ~11 and 37 years, shorter than our estimates of τ_14C_ (67–511 years), but matching those of τ_i_ (5–29 years). As our samples correspond to the top 20 cm of mineral soil, and τ_i_ is based on laboratory soil incubations, our results suggest that at least some ESMs focus on the turnover of the most labile SOC.

Radiocarbon and incubation-based turnover times τ_14C_ and τ_i_ were not strongly correlated (Pearson = 0.40, *p* < 0.01; Fig. 2), suggesting that the OC fractions these estimates reflect may be influenced by different drivers of SOC dynamics. Lastly, part of this discrepancy may be also related to the presence of pyrogenic organic carbon (PyOC). For example, data on PyOC by hydrogen pyrolysis on bulk soil samples of the 54 sites^36^, show that the two extreme points, with large τ_14C_ (~380 years) but low τ_i_ (~6 years) (Fig. 2), lie at the highest end of the range of probability distribution of PyOC (decile 7^th^ and 10^th^, Fig. S1). When we exclude these two sites from the analysis, the overall trend approaches the curvilinear relationship between mean residence time determined by ^14^C dating and by a ^13^C C_3_–C_4_ crop experiment in a study by Paul *et al*.^37^. The authors of this study concluded that the differences between the two turnover time estimations were because they respectively reflected the dynamics of a slow versus an active pool.

### Regional drivers of SOC turnover times

We did not find an effect of site temperature on τ_14C_ (Tables 1 and S1). Field warming experiments show a direct relationship between temperature and the short-term turnover of SOC, but there is evidence that this increase is ephemeral and that with sustained warming, the CO_2_ loss from soil tends to decline to ‘prior warming’ levels within a few years (≥10 years)^38–40^. Several mechanisms can explain thermal acclimation: (1) the depletion of microbially accessible OC pools together with the low sensitivity of an older pool^41–43^; (2) increased OC inputs and outputs^29^ and (3) the thermal acclimation of the microbial community (i.e. physiology, biomass, community composition) and enzymatic activities^44,45^. As for temperature, site moisture did not significantly influence τ_14C_ (Tables 1 and S1), indicating that acclimation might have also occurred, or that, over long timescales, τ_14C_ was not differently affected by the moisture regimes considered in this study. As such, these results suggest that at the regional scale, the influence of climate on the decadal-centennial turnover of SOC is not apparent, implying that the effect of a changing climate might be similar across climatic gradients.

**Table 1:** Results of regression analyses to investigate the influence of climate on τ_14C_, τ_i_ and DOC.

SOC dynamics	ANOVA structure	Significance of drivers					
		BGR	MI	TI	MI × TI	PCo1	PCo2
log τ_14C_	BGR + **MI × TI** + PCo1 + PCo2	*	n.s.	n.s.	n.s.	.	.
	BGR + **TI × MI** + PCo1 + PCo2	*	n.s.	n.s.	n.s.	.	.
log τ_i_	BGR + **MI × TI** + PCo1 + PCo2	***	**	n.s.	n.s.	***	n.s.
	BGR + **TI × MI** + PCo1 + PCo2	***	**	n.s.	n.s.	***	n.s.
DOC	BGR + **MI × TI** + PCo1 + PCo2	.		n.s.	n.s.	***	.
	BGR + **TI × MI** + PCo1 + PCo2	.	n.s.	n.s.	n.s.	***	.

Soil organic carbon (SOC) dynamics: τ_14C_ and τ_i_ are respectively the log-transformed ^14^C and incubation-based turnover times (years). DOC is the cumulative dissolved organic carbon produced during the 181-day incubation relative to the total organic carbon content of the mineral bulk soil (0–20 cm depth) at the beginning of the experiment (g kg^−1^OC). Drivers: BGR (biogeographical region), MI (site moisture two-level categorical variable: moist, dry), TI (site temperature two-level categorical variable: warm, cold), MI × TI (interaction of moisture and temperature or vice versa), PCo1 and PCo2 (each of the two orthogonal variables obtained by principal coordinates analysis that aggregate soil properties and landform-related variables). Symbols refer to p-values: ***<0.001, **<0.01, *<0.05, <0.1, ‘n.s.’ not significant. Crossed out values indicate that significance is not meaningful because it is not consistent across the two model structures tested for each metric of SOC dynamics. For extended results, refer to Table S1.

Although τ_i_ is assumed to be linked to climate and land cover type^46,47^, and despite it captured the dynamics of the most labile SOC fraction (Fig. 3), it was not significantly explained by site temperature (Tables 1 and S1). Therefore, either temperature plays a limited effect on τ_i_, or soils from cold sites are more sensitive to warming than those from warm sites, thus masking the effect of *in-situ* site temperature on the CO_2_ evolved during the incubation^48^. Acclimation processes might have also buffered the response of τ_i_ to site temperature; however, as τ_i_ averages ~10 years, the time available for acclimation to have occurred is shorter than in the case of τ_14C_, which averages ~172 years. Overall, these results indicate that at the regional scale, the influence of site temperature on the yearly-decadal turnover of SOC is not evident and that the net effect of warming might be similar across temperature gradients. Furthermore, τ_i_ was significantly related to site moisture (Tables 1 and S1) thus, the water balance appears to be more important than temperature for the turnover of the most labile SOC^7,29^. Our data reveals shorter τ_i_ (i.e. higher specific respiration) in dry sites than in moist sites. In the laboratory, when soils were under optimum conditions for decomposition, SOC that had accumulated in the field under dry conditions was mineralised quickly. This pattern evidences that in the field SOC mineralisation was predominantly limited by water availability, and manifests higher sensitivity of enzymes to moisture in dry sites^49^.

**Figure 4:**
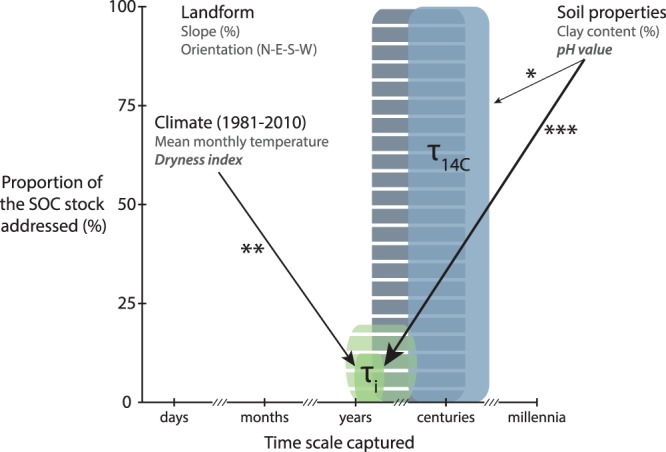
Schematic synthesis of the scope of ^14^C (τ_14C_) and incubation-based (τ_i_) turnover times. Full colours represent the breadth of this study and patterns the outlook when considering comparable studies on temperate forest topsoils^77–79^. We illustrate the drivers of soil organic carbon (SOC) dynamics considered in this study and highlight in italic, those that significantly explained turnover times. Stars and arrows’ thickness represent the strength of these significances. Significance code of p-values: ***<0.001, **<0.01, *<0.05.

The aggregated indicator of soil properties and landform PCo1 explained a significant part of the variation of τ_i_ and τ_14C_ (Tables 1 and S1). Further analyses revealed that soil pH was the underlying driver of τ_i_ and, to a lesser extent, of τ_14C_ (Tables 2, S2 and S3). Soil pH is involved in many processes affecting SOC availability and stabilisation and, it is expected that turnover times become shorter with increasing pH values^50^. The influence of site moisture status on τ_i_ but not on τ_14C_, together with the stronger influence of pH on τ_i_ than on τ_14C_, contribute to explain the lack of correlation between both turnover times (Fig. 2). These results confirm the occurrence of a spatio-temporal gap between the two SOC fractions these turnover times represent and demonstrate that drivers do not equally govern their dynamics (Fig. 4). Additionally, although in temperate forest soils pH is closely linked to the parent material^51^, we observed a significant but weak correlation between water balance and soil pH (Pearson = −0.35, *p* < 0.05), highlighting the influence of climate on determining soil pH at the global scale^52^.

**Table 2:** Results of regression analyses to investigate the influence of soil properties and landform on τ_14C_, τ_i_ and DOC.

SOC dynamics	ANOVA structure	Significance of drivers			
		pH	Clay	Slope	Orientation
log τ_14_	Driver + BGR + MI × TI	*	n.s.	n.s.	n.s.
	BGR + Driver + MI × TI	*	n.s.		n.s.
	BGR + MI × TI + Driver	*	n.s.		n.s.
log τ_i_	Driver + BGR + MIxTI	***	***	n.s.	
	BGR + Driver + MI × TI	***	***	n.s.	n.s.
	BGR + MI × TI + Driver	***	***	n.s.	n.s.
DOC	Driver + BGR + MI × TI	*	***	*	n.s.
	BGR + Driver + MI × TI	*	***	*	n.s.
	BGR + MI × TI + Driver	**	***	*	n.s.

Soil organic carbon (SOC) dynamics: τ^14^C and τi are respectively the log-transformed ^14^C and incubation-based turnover times (years). DOC is the cumulative dissolved organic carbon produced during the 181-day incubation relative to the total organic carbon content of the bulk mineral soil (0–20 cm depth) at the beginning of the experiment (g kg^−1^OC). Drivers: pH, clay (%), slope (%) and orientation (degrees). Symbols refer to p-values: ***<0.001, **<0.01, *<0.05, <0.1, ‘n.s.’ not significant. Crossed out values indicate that significance is not meaningful because it is not consistent across the three model structures tested for each indicator of SOC dynamics. For extended results, refer to Table S2.

### Regional drivers of DOC leaching

In the incubation assay, the cumulative DOC leached only accounted for ~1% of total OC losses (i.e. CO_2_ plus DOC). In agreement with results of previous studies, neither site temperature nor moisture emerged as significant drivers of this flux^53,54^ (Tables 1 and S1). Our analyses revealed clay content, pH and site slope as strong controls of DOC production. However, as differences in mineralogy often go along with changes in soil pH^55^, further statistical analyses showed that of the two covarying variables, clay and pH (Pearson = 0.39, *p* < 0.05), clay was driving the release of DOC (Tables 2, S2 and S3). We found lower DOC losses at higher clay contents, demonstrating the importance of organomineral interactions for the stabilisation of soil organic matter^56^.

With respect to the site slope, even though laboratory experiments are not able to represent many mechanisms that affect DOC fluxes under field conditions, such as the spatio-temporal variability of water transport, we found that steeper sites yielded smaller DOC amounts. We explain this result by a larger water run-off at steeper sites that limits the accumulation of soluble OC in the profile. Finally, despite organic substrates must be in dissolved form for enzymatic degradation, we did not find a correlation between DOC leaching and τ_i_ (Fig. S2). Results from other studies have also indicated that DOC production is not directly related to SOC turnover^53,54,57^, probably because only a small fraction of the total DOC is readily bio-available^58^.

To conclude, to our knowledge this is the first study on drivers of SOC dynamics that combines radiocarbon and incubation data adopting an experimental framework that statistically de-correlates the effects of temperature and moisture over a large network of sites. Our results suggest that ^14^C and incubation-based turnover times primarily capture the dynamics of an older, stabilised and a younger, readily available SOC fraction, respectively. Climatic controls did not affect the long-term ^14^C-based turnover time, challenging the SOC turnover mechanisms embodied in carbon models in which temperature and moisture are critical for decomposition kinetics. Nevertheless, for the shorter-term incubation-based turnover time, encompassing the decomposition of recently fixed carbon, the legacy effect of site moisture prevailed over the one of temperature. Further, our data revealed a prevailing control of soil pH over both turnover times. The production of DOC was independent of the incubation-based turnover time and neither controlled by climatic drivers, but by clay content. Our data endorses the action of soil physicochemical properties (e.g. pH, clay content) and landform as key drivers of SOC dynamics.

## Methods

### Selection of study sites

We followed a stepwise procedure to select the 54 study sites so that the effects of biogeographic region, site temperature, site moisture, and the aggregated soil properties and landform-related variables (PCo1, PCo2) were near-orthogonal (Fig. S3).

A common limitation of studies on the drivers of SOC dynamics is the co-variation of controls in time and/or space. This makes partitioning their relative influences challenging^13,14^. For example, if we take a set of random samples, it would be very difficult to statistically distinguish between the effect of decreasing temperature and increasing moisture, as in the temperate zone colder sites also tend to be moister. The knowledge-driven selection of study sites for soil research is not new^59^, but its application is still far from common practice. In this work, we developed a statistics-based strategy to select, from the soil database of the Swiss Federal Institute for Forest, Snow and Landscape Research (WSL)^15^, a set of 54 sites spread over Switzerland with minimised co-variation of the putative drivers (i.e. maximising orthogonality/statistical independence). The main hypothesis of this study is that sites air temperature and moisture play a primary role in SOC dynamics, followed by soil properties and landform. Accordingly, the stepwise implementation of the selection strategy was performed as follows with the R software (version 3.3.2)^60^:

1. **Definition of the exhaustive population within the study area**. By May 2014, the WSL soil database contained data on 1,050 profiles covering the main biogeoregions (i.e. Jura, Midland, Pre-Alps, Alps and Southern Alps)^16^ and a vast range of climatic conditions. 3% of the soil profiles in the database belong to the Long-term Forest Ecosystem Research Programme (LWF, German acronym). LWF sites are of special interest due to the sustained and intensive research activities and availability of data. Studies conducted in forested areas have shown that strong topographic gradients are an important control of erosion and deposition patterns^61,62^. For this reason, to assume that C losses due to erosion are negligible relative to the rate of SOC mineralisation, we excluded sites, excepting LWF sites, with slopes larger than 50% (26.57 degrees). Once we applied this filter, the working database counted 709 entries.
2. **Definition of the putative drivers**. To represent climate, we included sites temperature (1981–2010 average air mean monthly temperature) and moisture (1981–2010 dryness index). The dryness index is a proxy to site moisture status based on the cumulative number of dry months. A dry month is one on which the precipitation of the month is smaller than its potential evapotranspiration (Penman)^63^. For soil properties and landform, we reduced the pH value, clay content (%), slope (%) and orientation (N-E-S-W) to two orthogonal variables (PCo1, PCo2) by principal coordinates analysis^18,64^. For the selection of the sites, all data was sourced from the WSL soil database.
3. **Partition of the population into four equiprobable categories based on main hypothesised drivers**. As we hypothesise that climate plays a primary role in SOC dynamics, we partitioned the population into four equiprobable categories based on the median value of site temperature and moisture.
4. **Spatial distribution of the population based on biogeographical regions**. Swiss biogeographical regions are the product of a statistical classification based on floristic and faunal distribution patterns, thus they are also connected to geophysical characteristics. To ensure the spatial distribution of the study sites, we subdivided each climatic category upon biogeographical regions. Some combinations of climate-biogeographical region did not occur or were under-represented. For example, we did not find the combination of moist and warm sites in the Alpine region or moist sites in the Southern Alps region.
5. **Partition of the population into four categories based on secondary hypothesised drivers**. We reduced pH, clay (%), slope (%) and orientation (N-E-S-W) into two variables (PCo1 and PCo2) product of a principal coordinates analysis performed on the population dataset (PCoA; also known as classical or metric multidimensional scaling)^18,64^. PCoA is an ordination method that attempts to position objects in a Euclidean space of reduced dimensionality while preserving their distance relationships of dissimilarity (or similarity)^64^. As a result, in the graphical representation of the Euclidean space, the further are the sites plotted to each other, the more dissimilar they are and vice-versa.
6. **Non-automated selection of study sites from two-dimensional Euclidean spaces defined by PCo1 and PCo2**. To select the 54 sites, we positioned the data from each climate-biogeographical region combination into two-dimensional Euclidean spaces defined by PCo1 and PCo2. From here, we selected equidistant sites from the origin of coordinates. Due to missing points, some combinations were not sampled. These gaps are expected to have an only minor influence on the regression analyses because they are spread rather than clustered over the full range of potential combinations.

### Field campaign

Between July and September 2014, we collected from each of the 54 sites three mineral soil samples from three non-overlapping areas of a 40 × 40 m^2^ plot immediate to the original WSL soil profile. Each of these samples was a composite product of mixing eight 0–20 cm depth soil cores collected with a 5 cm diameter Humax corer. This sampling strategy enabled us to account for soil spatial variability. After collection, we transported the soils in portable fridges to the laboratories where we sieved them by hand (≤2 mm) and stored them at 3.5 °C until further use.

### Laboratory soil incubation

In February 2015, we incubated fresh soil samples (sieved to ≤2 mm; 40 g equivalent dry mass; adjusted to 0.8 g cm^−3^ bulk density) in sterilised glass microlysimeters^20^ which permitted the flushing of the samples during the incubation to yield soil water extracts. Microlysimeters were placed into 2-litre airtight glass jars that contained 20 ml of distilled water in a separate beaker to ensure the headspace was moist. After a 10-day pre-incubation, run under the same conditions as the incubation assay, we measured SOC mineralisation for 181 days while keeping soil moisture (−20 kPa) and temperature (25 °C) constant. We adjusted soil moisture with a suction pump and controlled it periodically by weighing the samples. Our goal was not to estimate the turnover time of soils in the field but to establish comparable conditions close to optimum for microbial activity driving SOC mineralisation. As the laboratory incubation ran at 25 °C, it meant an average temperature increase of 17.3 °C compared to field temperatures (range of temperature increase from 13.2–24.0 °C). We intentionally applied this large increase in temperature to override different transient warming responses that might have occurred when warming soil from cold and warm sites^48^. The CO_2_-C product of the mineralisation of SOC was captured in 20 ml NaOH (1M) traps placed into the 2-litre glass jars. Subsequently, the amount of C trapped was determined by the change of conductivity of the NaOH^65^. The amount of dissolved organic carbon (DOC) in the soil water extracts was determined by TOC analysis (DIMA TOC-2000 Dimatec, Essen, Germany). From the beginning of the experiment, NaOH traps were replaced for fresh ones on days 4, 13, 30, 63, 92, 121, 150, and 181. On days 4, 13, 30, 63, 121, and 181, each microlysimeter was also leached with 30 ml of a nutrient solution^66^ without N or P. Subsequently, after equilibration for 30 minutes, a suction of −20 kPa was applied for 25 minutes to the systems. In a previous study, we verified that leaching and the potential accumulation of mineral nitrogen (NO^−^_2_, NO^−^_3_, NH^+^_4_) during incubation does not bias SOC mineralisation^20^. DOC samples were filtered (1.6 µm MGA glass microfibre, Sartorius) before analysis. Incubation data (i.e. SOC mineralised, DOC) were normalised relative to the total organic carbon content of the bulk soil at the beginning of the experiment.

### Radiocarbon measurements

In this study, we measured the radiocarbon content of (1) bulk soil organic carbon and (2) CO_2_–C evolved from bulk soil. Measurements were performed on a MIni CArbon DAting System (MICADAS, Ionplus, Switzerland)^19^ featuring a gas ion source^67^ and coupled to an Elemental Analyser (EA vario MICRO cube, Elementar, Germany) at the Laboratory of Ion Beam Physics, ETH Zurich.

1. We measured the radiocarbon signature of bulk soil organic carbon on mixtures of the three composite samples collected at each of the 54 study sites. Prior analysis, we acidified (HCl 37% Trace-Metal purity, 60 °C, 72 h) and neutralized (NaOH pellets, 72 h, 60 °C) soils to remove potential inorganic carbon. Samples were combusted in the EA and the gas generated measured in the MICADAS.
2. We measured the radiocarbon signature of the CO_2_ evolved on mixtures of the NaOH traps of the three composites containing the CO_2_-C evolved during the last 31 days of the 181-day incubation. We selected the last sampling period of the incubation in order to minimise the effect of soil disturbance as affected by the incubation preparation. After mixing, we acidified the NaOH samples with H_3_PO_4_ (85% purity) and measured the gas generated in the MICADAS. Incubated blanks (i.e. jars without soil) were used to correct the radiocarbon signatures and to incorporate a total uncertainty based on a mixing model assuming constant contamination^68^.

Radiocarbon values are reported in Δ^14^C. We corrected the standards OX-I (SRM-4990, NIST) and OX-II (SRM-4990C, NIST) for the decay undergone from 1950 to 2014, year of field sampling.

### ^14^C and incubation-based turnover times

Various observational techniques have been developed to evaluate SOC dynamics and the magnitude and variability of these estimations is largely method dependent^28,37,69,70^. In fact, one single satisfactory technique does not exist and each of them may be suited to capture the dynamics of SOC at different timescales^70,71^. Turnover time is a metric of SOC dynamics that at steady-state often refers to the ratio of total carbon stock to input or output flux^72^. In a homogeneous system at steady-state, it indicates the time it would take for a reservoir to empty if there were no further inputs^73^. When data are not interpreted adequately, the assumption of a single homogeneous pool can lead to misconception. Soils with similar mean turnover time may present distinct distributions of turnover among pools, thus running the risk of overlooking the dynamics of smaller temperature-sensitive labile SOC fractions^74^. We acknowledge that soils constitute a heterogeneous mixture of organic C compounds cycling along a continuum of timescales (i.e. from minutes to millennia). Therefore, although in this study we assumed system homogeneity, we calculated two separate estimates of turnover time based on bulk soil ^14^C measurements (τ_14C_) and on a 6-month laboratory soil incubation (τ_i_), capturing the dynamics of contrasting ‘fractions’ of the SOC continuum.

1. ^**14**^**C-based turnover time**. ^14^C data is applied in open systems to estimate the rate of exchange of carbon between reservoirs^34,72^. This technique takes advantage of the ^14^C produced in the explosion of thermonuclear weapons during the early 1960s^75^. The degree to which bomb ^14^C is found in SOC provides a direct measure of the extent to which soils have incorporated C from the atmosphere^34^. In this work, we followed an approach that models a single homogeneous reservoir at steady-state, incorporating bomb ^14^C since 1950 and that does not account for lag times (i.e. time gap between C fixation in photosynthesis and release via soil microbial mineralisation)^21,72,73^. This method provides two possible solutions, but as our samples are 0–20 cm depth mineral soils, we always adopted the solution after the bomb ^14^C peak in 1964.

$${\Delta }^{14}{C}_{t+h}=k\,h\,{\Delta }^{14}{C}_{atm,t+h}+{\Delta }^{14}{C}_{t}(1-h(k+\lambda ))$$

Where:

- Δ^14^C is the soil sample radiocarbon content
- k is the SOC decomposition rate constant (year^−1^) and its inverse corresponds to the turnover time, τ = 1/k (year)
- h is the time step used in the calculation (1/12 for monthly time step)
- Δ^14^C_atm_ is the radiocarbon content of the atmospheric C
- t + h corresponds to the time for which the model runs (month)
- t refers to a previous time step (month)
- λ is the radiocarbon decay constant and corresponds to the inverse of Godwin mean life (1/8267 = 1.21 × 10^−4^ year^−1^)

2. **Incubation-based turnover time**. Under the assumptions of one homogenous pool, we calculated turnover time as the ratio of total C stock to input or output flux^22^.

### pH, total organic carbon (TOC) & other data

We measured soil pH (CaCl_2_) on 40 °C dried composite subsamples. Part of these subsamples was also milled and subjected to acid fumigation to quantify TOC by Elemental Analysis (vario MICRO cube, Elementar, Germany). The rest of data were sourced from the WSL soil database^15,76^.

### Data analysis

As the 54 study sites were selected to maximise the orthogonality of the drivers, their effects were independent in the sequential fitting of multiple linear regression models summarized by analysis of variance^17,18^. As Swiss biogeographical regions are indirectly connected to biogeophysical characteristics, which may covary and confound the effect of the drivers of interest, we introduced them as a blocking factor in the regression models. Analyses of τ_i_ exclude sites 6, 12 and 24. Sites 6 and 24 were removed because NaOH traps became saturated (i.e. ≥80% of their uptake capacity was reached). By removing saturated samples, we guarantee a linear relationship between the conductivity of the alkali solution and avoid isotopically fractionated samples. Although the inclusion or exclusion of site 12 did not determine what drivers emerged significant, we rejected this site to improve the distribution of the residuals. On the other side, analyses of τ_14C_ exclude site 40 (Eschenbach, Luzern, Midland) because we consider that the large turnover time obtained for this site (~1140 years) was due to the accidental collection of soil >20 cm depth. Both, τ_i_ and τ_14C_ were log transformed to improve the distribution of residuals. We used DOC and drivers data untransformed. Analyses were performed with the R software (version 3.3.2)^60^.

## Supplementary information

Supplementary information

## Data Availability

Data necessary to interpret, replicate and build upon this study are shared via a public repository (DOI 10.5281/zenodo.2526673). For access to the complete forest soil database of the Swiss Federal Institute for Forest, Snow and Landscape Research (n = 1050), you may contact S. Zimmermann (stephan.zimmermann@wsl.ch) or L. Walthert (lorenz.walthert@wsl.ch). List of figures that contain associated raw data: Figs 1–3 and S1–S4.
