# Supplementary material for: Temperature and moisture are minor drivers of regional-scale soil organic carbon dynamics
Source: Sci Rep. 2019 Apr 23;9:6422. doi: 10.1038/s41598-019-42629-5 (PMC6478928; doi:10.1038/s41598-019-42629-5)
Supplement: Supplementary file 1 — Supplementary information [file 41598_2019_42629_MOESM1_ESM.docx]

**Temperature and moisture are minor drivers of regional-scale soil organic carbon dynamics**

B. González-Domínguez*^1,2^, P.A. Niklaus^2^, M.S. Studer^1^, F. Hagedorn^3^, L. Wacker^4^, N. Haghipour^4,5^, S. Zimmermann^3^, L. Walthert^3^, C. McIntyre^4,5,6^, S. Abiven^1^

1-Department of Geography, Soil Science and Biogeochemistry Unit, University of Zurich (UZH), Winterthurerstrasse 190, 8057 Zurich, Switzerland

2-Department of Evolutionary Biology and Environmental Studies, University of Zurich (UZH), Winterthurerstrasse 190, 8057 Zurich, Switzerland

3-Forest Soils and Biogeochemistry, Swiss Federal Institute for Snow, Forest and Landscape Research (WSL), Zürcherstrasse 111, 8903 Birmensdorf, Switzerland

4-Department of Physics, Laboratory of Ion Beam Physics, Swiss Federal Institute of Technology (ETH), Otto-Stern-Weg 5, 9083 Zurich, Switzerland

5-Institute of Geology, Department of Earth Sciences, Swiss Federal Institute of Technology (ETH), Sonneggasse 5, 8092 Zurich, Switzerland

6-AMS Laboratory, Scottish Universities Environmental Research Centre (SUERC), Rankine Avenue, G75 0QF, East Kilbride, UK


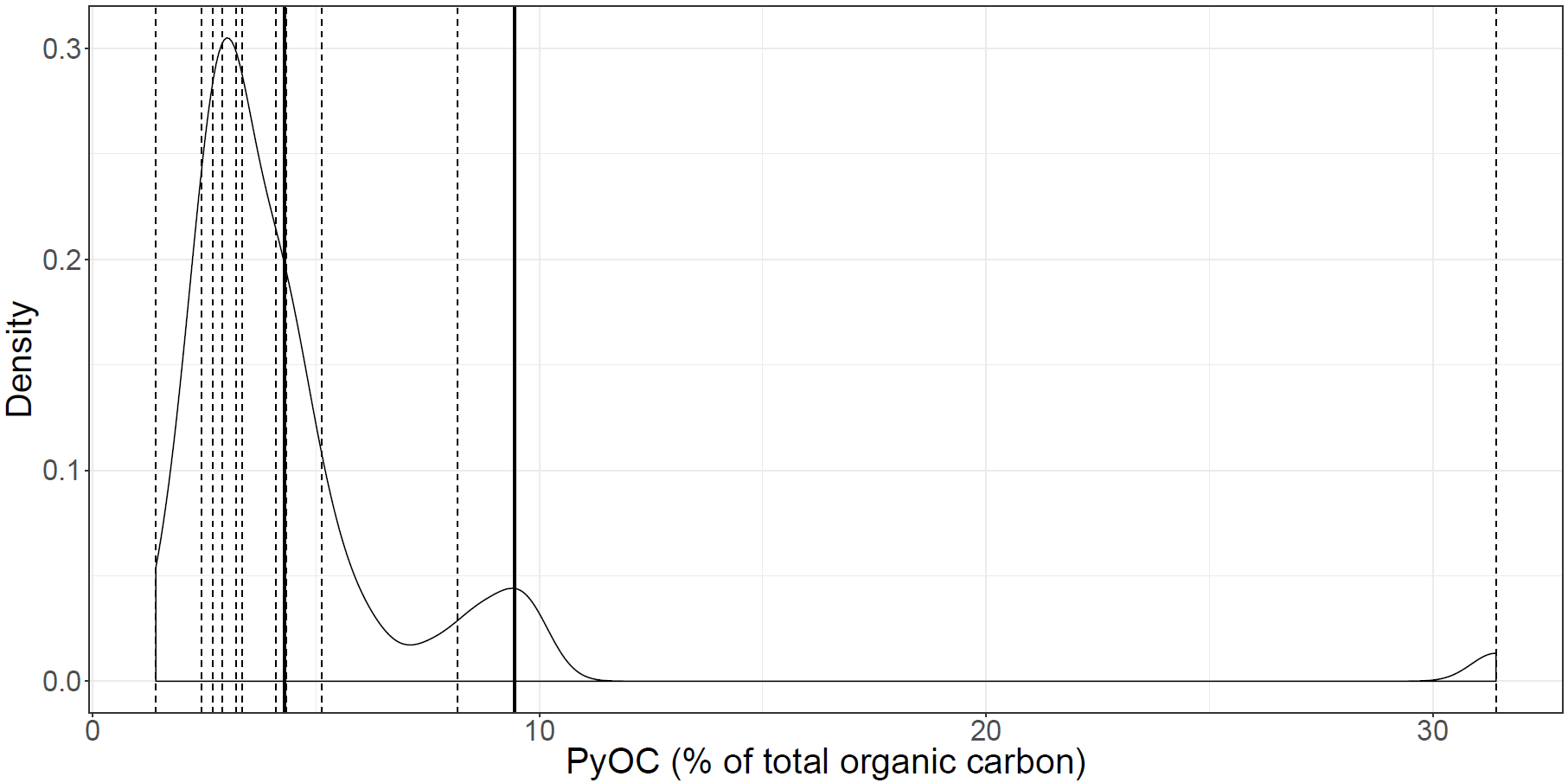


Figure S 1 – Range of probability distribution of pyrogenic organic carbon (PyOC, % of total organic carbon) estimated by hydrogen pyrolysis^1^ on the 54 study sites (Figure 1). Discontinuous lines are deciles and solid lines are the two samples at the highest end of τ_14C_ (~380 years) but at the lowest end of τ_i_ (~6 years) in Figure 2.


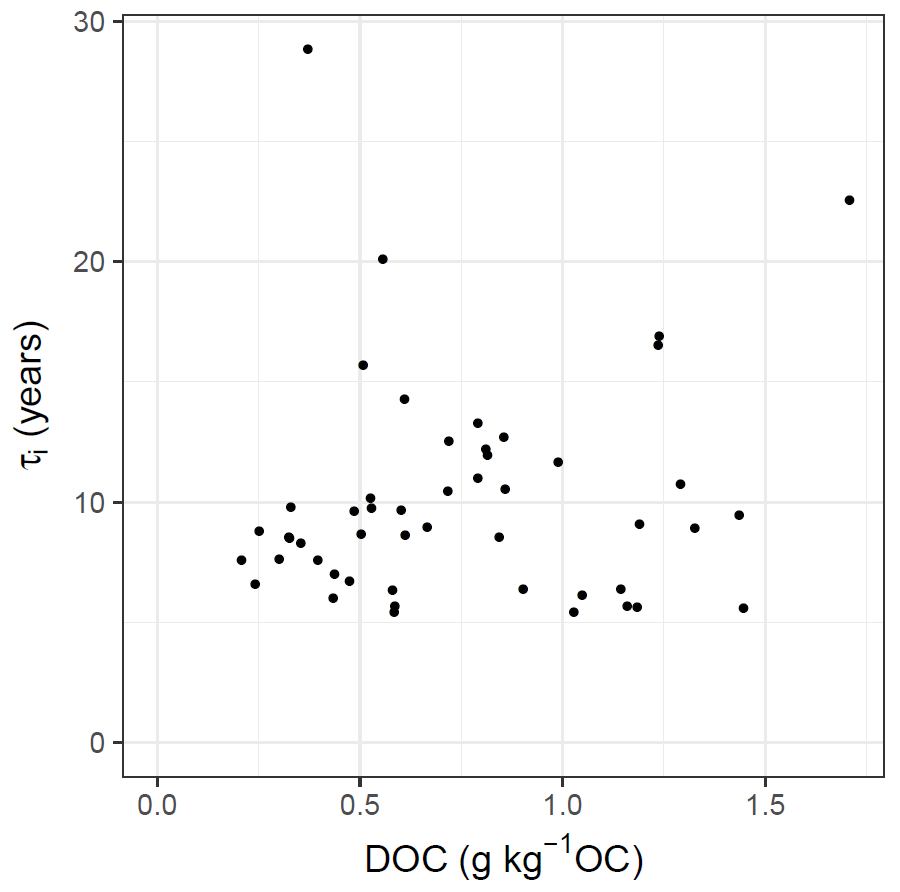


Figure S 2 – No significant correlation between the incubation-based turnover time τ_i_ and the cumulative dissolved organic carbon (DOC) leached during the 181-day incubation.


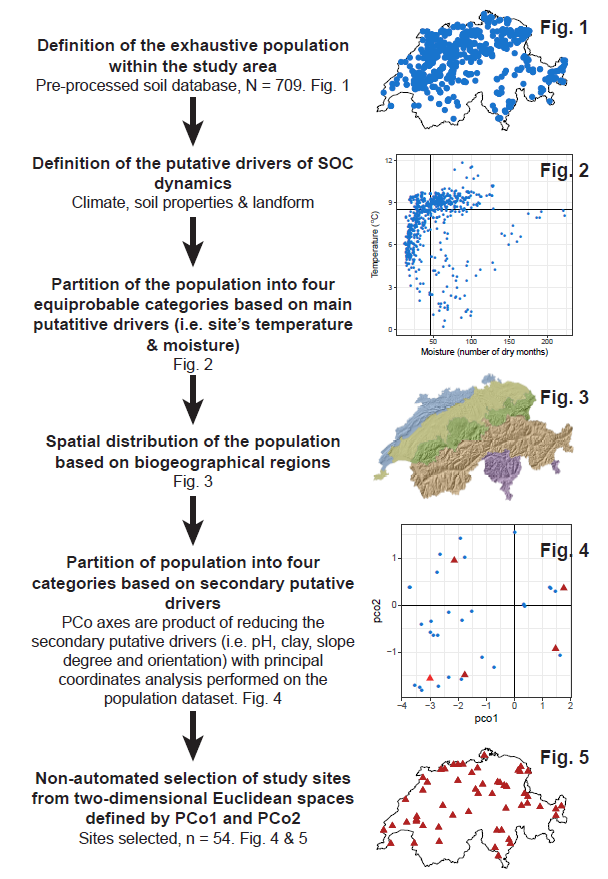


Figure S 3 – Schematic representation of the stepwise statistics-based selection of the 54 study sites.


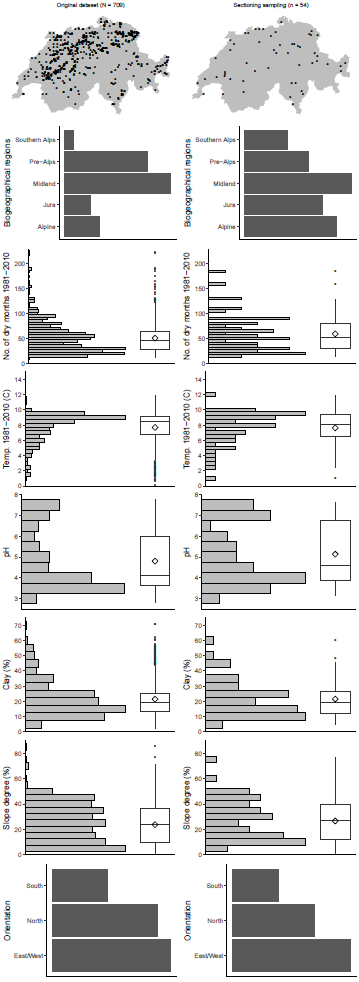


Figure S 4 – Distribution of drivers’ data in the forest soils database of the Swiss Federal Institute for Snow, Forest and Landscape Research (WSL) (N remaining = 709) and the 54 study sites. Sites with slopes > 50%, excepting those part of the Long-term Forest Ecosystem Research Programme, are not represented.

Table S 1 – Extended results of regression analyses to investigate the influence of climate on τ_14C_, τ_i_ and DOC. Soil organic carbon (SOC) dynamics: τ_14C_ and τ_i_ are respectively the log-transformed ^14^C and incubation-based turnover times (years). DOC is the cumulative dissolved organic carbon produced during the 181-day incubation relative to the total organic carbon content of the bulk soil at the beginning of the experiment (g kg^-1^OC). Drivers: BGR (biogeographical region), MI (site moisture two-level categorical variable: moist, dry), TI (site temperature two-level categorical variable: warm, cold), MI×TI (interaction of moisture and temperature or vice versa), PCo1 and PCo2 (each of the two orthogonal variables obtained by principal coordinates analysis that aggregate soil properties and landform-related variables). Residual degrees of freedom are 43 for τ_14C_, 41 for τ_i_, and 44 for DOC. ‘n.s.’ not significant. No meaningful significances are those not consistent across the three model structures tested for each indicator of SOC dynamics. For a simplified table, see Table 1.

| SOC dynamics | ANOVA structure | Effect BGR | | | Effect MI | | | Effect TI | | | Effect MI×TI | | | Effect PCo1 | | | Effect PCo2 | | |
| --- | --- | --- | --- | --- | --- | --- | --- | --- | --- | --- | --- | --- | --- | --- | --- | --- | --- | --- | --- |
|  |  | F | df | p-value | F | df | p-value | F | df | p-value | F | df | p-value | F | df | p-value | F | df | p-value |
| log τ_14C_ | BGR + **MI×TI** + PCo1 + PCo2 | 2.799 | 4 | 0.038 | 0.290 | 1 | n.s. | 0.074 | 1 | n.s. | 0.072 | 1 | n.s. | 2.945 | 1 | 0.093 | 3.615 | 1 | 0.064 |
|  | BGR + **TI×MI** + PCo1 + PCo2 | 2.799 | 4 | 0.038 | 0.351 | 1 | n.s. | 0.013 | 1 | n.s. | 0.072 | 1 | n.s. | 2.945 | 1 | 0.093 | 3.615 | 1 | 0.064 |
| log τ_i_ | BGR + **MI×TI** + PCo1 + PCo2 | 7.398 | 4 | ≤ 0.001 | 8.198 | 1 | 0.007 | 0.609 | 1 | n.s. | 0.254 | 1 | n.s. | 31.543 | 1 | ≤ 0.001 | 1.479 | 1 | n.s. |
|  | BGR + **TI×MI** + PCo1 + PCo2 | 7.398 | 4 | ≤ 0.001 | 8.772 | 1 | 0.005 | 0.035 | 1 | n.s. | 0.254 | 1 | n.s. | 31.543 | 1 | ≤ 0.001 | 1.479 | 1 | n.s. |
| DOC | BGR + **MI×TI** + PCo1 + PCo2 | 2.084 | 4 | 0.099 | 3.416 | 1 | 0.071 | 1.315 | 1 | n.s. | 0.090 | 1 | n.s. | 34.588 | 1 | ≤ 0.001 | 3.420 | 1 | 0.071 |
|  | BGR + **TI×MI** + PCo1 + PCo2 | 2.084 | 4 | 0.099 | 2.250 | 1 | n.s. | 2.481 | 1 | n.s. | 0.090 | 1 | n.s. | 34.588 | 1 | ≤ 0.001 | 3.420 | 1 | 0.071 |

Table S 2 – Extended results of regression analyses to investigate the influence of soil properties and landform on τ_14C_, τ_i_ and DOC. Soil organic carbon (SOC) dynamics: τ_14C_ and τ_i_ are respectively the log-transformed ^14^C and incubation-based turnover times (years). DOC is the cumulative dissolved organic carbon produced during the 181-day incubation relative to the total organic carbon content of the bulk soil at the beginning of the experiment (g kg^-1^OC). Drivers: pH, clay (%), slope (%), orientation (degrees) and gradient, which integrates slope and orientation ($Gradient North=cos(orientation/(180\times\pi))\times slope$ and $Gradient East=sin(orientation/(180\times\pi))\times slope$. Drivers also in the models but with significances not reported: BGR (biogeographical region), MI (site moisture two-level categorical variable: moist, dry), TI (site temperature two-level categorical variable: warm, cold). In the model structure, MI×TI refers to the testing of MI, TI and their interaction. Residual degrees of freedom are 44 for τ_14C_, 42 for τ_i_, and 45 for DOC. ‘n.s.’ not significant. No meaningful significances are those not consistent across the three model structures tested for each indicator of SOC dynamics. For a simplified table, see Table 2. As clay (%) and pH are correlated, we tested various model structures to de-confound their single effects on τ_i_ and DOC (see Table S 3).

| SOC dynamics | ANOVA structure | Effect pH | | | Effect Clay | | | Effect Slope | | | Effect Orientation | | | Effect Gradient North | | | Effect Gradient East | | |
| --- | --- | --- | --- | --- | --- | --- | --- | --- | --- | --- | --- | --- | --- | --- | --- | --- | --- | --- | --- |
|  |  | F | df | p-value | F | df | p-value | F | df | p-value | F | df | p-value | F | df | p-value | F | df | p-value |
| log τ_14_ | Driver + BGR + MI×TI | 6.435 | 1 | 0.015 | 0.179 | 1 | n.s. | 0.160 | 1 | n.s. | 0.682 | 1 | n.s. | 2.445 | 1 | n.s. | 0.048 | 1 | n.s. |
|  | BGR + Driver + MI×TI | 4.337 | 1 | 0.043 | 0.035 | 1 | n.s. | 4.138 | 1 | 0.048 | 0.050 | 1 | n.s. | 2.391 | 1 | n.s. | 0.177 | 1 | n.s. |
|  | BGR + MI×TI + Driver | 5.165 | 1 | 0.028 | 0.045 | 1 | n.s. | 4.458 | 1 | 0.040 | 0.140 | 1 | n.s. | 2.798 | 1 | n.s. | 0.066 | 1 | n.s. |
| log τ_i_ | Driver + BGR + MI×TI | 83.952 | 1 | ≤ 0.001 | 18.172 | 1 | ≤ 0.001 | 0.005 | 1 | n.s. | 4.279 | 1 | 0.045 | 0.113 | 1 | n.s. | 2.464 | 1 | n.s. |
|  | BGR + Driver + MI×TI | 60.041 | 1 | ≤ 0.001 | 13.539 | 1 | ≤ 0.001 | 2.079 | 1 | n.s. | 2.308 | 1 | n.s. | 0.084 | 1 | n.s. | 2.380 | 1 | n.s. |
|  | BGR + MI×TI + Driver | 50.830 | 1 | ≤ 0.001 | 14.987 | 1 | ≤ 0.001 | 1.730 | 1 | n.s. | 1.210 | 1 | n.s. | 0.351 | 1 | n.s. | 1.028 | 1 | n.s. |
| DOC | Driver + BGR + MI×TI | 6.094 | 1 | 0.017 | 30.981 | 1 | ≤ 0.001 | 5.962 | 1 | 0.019 | 0.308 | 1 | n.s. | 1.042 | 1 | n.s. | 0.308 | 1 | n.s. |
|  | BGR + Driver + MI×TI | 7.141 | 1 | 0.010 | 31.971 | 1 | ≤ 0.001 | 6.237 | 1 | 0.016 | 0.069 | 1 | n.s. | 0.320 | 1 | n.s. | 0.133 | 1 | n.s. |
|  | BGR + MI×TI + Driver | 11.067 | 1 | 0.002 | 32.965 | 1 | ≤ 0.001 | 6.122 | 1 | 0.017 | 0.061 | 1 | n.s. | 0.137 | 1 | n.s. | 0.131 | 1 | n.s. |

Table S 3 – As clay (%) and pH are correlated (Spearman = 0.45, *p* = 0.001), we tested various model structures to de-confound their single explanatory effects on τ_i_ and DOC. Soil organic carbon (SOC) dynamics: τ_i_ corresponds to log-transformed incubation-based turnover times (years). DOC is the cumulative dissolved organic carbon produced during the 181-day incubation relative to the total organic carbon content of the bulk soil at the beginning of the experiment (g kg^-1^OC). Drivers: BGR (biogeographical region), MI (site moisture two-level categorical variable: moist, dry), TI (site temperature two-level categorical variable: warm, cold), pH and clay (%). Drivers also in the models but with significances not reported: BGR (biogeographical region), MI (site moisture two-level categorical variable: moist, dry), TI (site temperature two-level categorical variable: warm, cold). In the model structure, MI×TI refers to the testing of MI, TI and their interaction. ‘Structure driver’ refers to the order in which clay and pH were introduced into the regression linear models. As these models are sequential, the explanatory power of the first variable is subtracted before it is fitted for the next variable. For example, in the case of ‘Clay+pH’, the model is first fitted for clay and then for pH, and thus, we obtain the explanatory power of pH when we remove the ‘influence’ of clay. For this reason, in this case, the relevant significance is the one of pH. Residual degrees of freedom are 44 for τ_14C_, 42 for τ_i_, and 45 for DOC. ‘n.s.’ not significant. No meaningful significances are those not consistent across the three model structures tested for each indicator of SOC dynamics.

| SOC dynamics | ANOVA structure | Effect pH  (structure: Clay+pH) | | | Effect Clay  (structure: pH+Clay) | | |
| --- | --- | --- | --- | --- | --- | --- | --- |
|  |  | F | df | p-value | F | df | p-value |
| log τ_i_ | Driver + BGR + MI×TI | 61.448 | 1 | ≤ 0.001 | 2.724 | 1 | n.s. |
|  | BGR + Driver + MI×TI | 44.195 | 1 | ≤ 0.001 | 3.152 | 1 | 0.083 |
|  | BGR + MI×TI + Driver | 32.922 | 1 | ≤ 0.001 | 4.379 | 1 | 0.043 |
| DOC | Driver + BGR + MI×TI | 0.738 | 1 | n.s. | 24.819 | 1 | ≤ 0.001 |
|  | BGR + Driver + MI×TI | 1.522 | 1 | n.s. | 25.107 | 1 | ≤ 0.001 |
|  | BGR + MI×TI + Driver | 4.149 | 1 | 0.048 | 22.954 | 1 | ≤ 0.001 |

**References of Supplementary Information**

1. Reisser, M. *et al.* Drivers of Pyrogenic Carbon content and its long lasting influence on Soil Organic Carbon in forest soils (in prep.). (2018).
